# CCR5 facilitates endothelial progenitor cell recruitment and promotes the stabilization of atherosclerotic plaques in ApoE^−/−^ mice

**DOI:** 10.1186/s13287-015-0026-0

**Published:** 2015-03-19

**Authors:** Zhongwen Zhang, Jianjun Dong, Corrinne G Lobe, Peiyun Gong, Ju Liu, Lin Liao

**Affiliations:** Department of Medicine, Shandong Provincial Qianfoshan Hospital, Shandong University, 16766 Jingshi Road, Jinan, Shandong 250014 China; Department of Medicine, Qilu Hospital of Shandong University, Wenhua Road, Jinan, 250012 China; Miami Mice Research Corp., 101 College Street, Toronto, Ontario M5G 1 L7 Canada; Medical Research Center, Shandong Provincial Qianfoshan Hospital, Shandong University, 16766 Jingshi Road, Jinan, Shandong 250014 China

## Abstract

**Introduction:**

Unstable atherosclerotic plaques are prone to rupture, which leads to atherothrombosis. Endothelial progenitor cells (EPCs) are bone marrow-derived precursor cells that may repair vascular injury in atherosclerosis. Chemokine (C-C motif) receptor 5 (CCR5) promotes mobilization of EPCs. In this study, we investigated the therapeutic potential of CCR5-overexpressing EPCs on plaque stabilization in an apolipoprotein E (ApoE)^−/−^ mouse model.

**Methods:**

The expression of CCR5 and its cognate ligand chemokine (C-C motif) ligand 5 (CCL5) was examined in atherosclerotic aortas of humans and mice by immunohistochemistry. Splenectomized ApoE^−/−^ C57BL/6 J mice fed a high-fat diet for 24 weeks were intravenously injected with EPCs transfected with CCR5 overexpression lentivirus. The recruitment of EPCs over the atherosclerotic plaques was evaluated by immunofluorescence. The content of lipid, smooth muscle cells, monocytes/macrophages, and endothelial cells in atherosclerotic plaques was assayed by specific immunostaining. The serum levels of atherosclerosis-related inflammatory factors in ApoE^−/−^ mice were measured by mouse atherosclerosis antibody array I.

**Results:**

CCR5 and CCL5 are highly expressed in atherosclerotic plaques in both humans and mice. The ApoE^−/−^ mice with CCR5-overexpressing EPC treatment demonstrated a more stable plaque formation with enhanced recruitment of EPC, reduced lipid, and macrophage content in the atherosclerotic plaques. CCR5-overexpressing EPC treatment also increased the content of endothelial cells and nitric oxide production in the plaques. In addition, the serum levels of interleukin-3 (IL-3), IL-5, IL-6, IL-13, CD40, and tumor necrosis factor-alpha and the plaque contents of IL-6 and matrix metalloproteinase-9 were reduced in mice with CCR5-overexpressing EPC treatment.

**Conclusions:**

These findings suggest that CCR5 is a novel therapeutic target in EPC treatment for stabilization of atherosclerotic plaques.

**Electronic supplementary material:**

The online version of this article (doi:10.1186/s13287-015-0026-0) contains supplementary material, which is available to authorized users.

## Introduction

Atherothrombotic events, such as myocardial infarction and strokes, are the most devastating clinical manifestations of atherosclerosis [1,2]. The leading cause of atherothrombosis is atherosclerotic plaque rupture [1,2], which is characterized by injury of endothelium and exposure of thrombogenic lipid core into the bloodstream. The current treatments are limited in their overall effectiveness. The lipid-lowering and anti-platelet drug treatments are not sufficient to stabilize vulnerable plaques, and intervention therapies might result in re-narrowing [3]. Thus, it is crucial to find new approaches to reduce atherosclerotic plaque rupture, and eventually reduce the disease burden.

Endothelial cells (ECs) play a crucial role in the formation and stabilization of atherosclerotic plaques [1-4]. High cholesterol, high blood pressure, or diabetes induces EC dysfunction and damages the integrity of the endothelium. Circulating low-density lipoprotein cholesterol crosses the damaged endothelium and accumulates in the wall of the artery, initiating the plaque formation [5]. During the development of atherosclerotic plaques, apoptosis of ECs over the plaques leads to enlargement of the lipid core, loss of collagen, and intimal inflammation [3,4]. Endothelial progenitor cells (EPCs) are a type of bone marrow (BM)-derived precursor cells that can differentiate *ex vivo* to an endothelial phenotype [3,4,6]. Upon EC dysfunction, EPCs from BM move into the circulation and replace the damaged cells [3,4,6-8]. However, mobilization of EPCs from BM to the atherosclerotic plaques is very limited in non-treatment conditions [9]. Thus, interventions improving EPC recruitment may present a novel strategy for plaque stabilization.

Chemokine receptor 5 (CCR5) is a member of the β-chemokine receptor family and a G-coupled seven-transmembrane chemokine receptor [10]. CCR5 is expressed in leukocytes and monocytes/macrophages [11]. Genetic inactivation of CCR5 is associated with the reduction of pro-atherogenic cytokines and the accumulation of monocytes/macrophages in atherosclerotic plaques [12,13]. CCR5’s cognate ligand chemokine ligand 5 (CCL5), also known as RANTES (regulated on activation, normal T cell expressed and secreted), is a member of the CC-chemokine family stored in and released from platelets and activated T cells [14]. CCL5 is upregulated in the injured vessels via activation by platelets during the process of atherosclerosis [9,15]. Increased expression of CCL5 on the surface-adherent platelets mediates trafficking of monocytes/macrophages into injured vessels by binding with its receptor CCR5 [10,12,13]. To date, the effects of CCR5 on the stability of atherosclerotic plaques have not been addressed. Recent studies reported that CCR5 mediates glomerular microvascular endothelial regeneration by stimulating the adhesion of BM-derived EPCs [14] and that inhibition of CCR5 expression reduces EPC recruitment during wound healing in mice [16]. Thus, we hypothesize that increased expression of CCR5 in EPCs may enhance the stability of plaques by stimulation of EPC mobilization and recruitment.

In this study, we examined the role of CCR5 in EPCs on atherosclerotic plaques by injection of CCR5-overexpressing EPCs into the ApoE^−/−^ mice and evaluation of plaque stability in these mice. We found that overexpression of CCR5 in EPCs enhanced the accumulation of EPCs over atherosclerotic plaques. In addition, CCR5-overexpressing EPC treatment decreased the macrophage content on advanced atherosclerotic plaques. Our findings indicate that overexpression of CCR5 improves EPC treatment for the stabilization of atherosclerotic plaques.

## Methods

### Animals

ApoE^−/−^ C57BL/6 J male mice were purchased from Vital River (Beijing, China). All animal studies were performed at the Animal Care Center of the Key Laboratory of Cardiovascular Remodeling and Function Research, Shandong University (Shandong, China). The animal experiments followed the Regulation of Animal Care Management of the Ministry of Public Health, People’s Republic of China (document 55, 2001) and were approved by the Ethical Committee of Shandong University.

### Splenectomy surgery

The spleens of the mice were dissected as previously described [17,18]. In brief, mice (8 to 10 weeks old) were anesthetized with intraperitoneal administration of 0.8% pentobarbital sodium (10 mg/kg body weight). An incision of 10 to 15 mm was made above the left abdomen of each mouse, and the spleen was removed after cauterizing the splenic arteries and venous supply. The mice were allowed to recover for 7 days before further treatment.

### Isolation and culture of mouse endothelial progenitor cells

EPCs were isolated from 8-week-old male C57 mice. In brief, whole BM cells were collected under sterile conditions from femur (and tibias) by flushing the shaft with phosphate-buffered saline (PBS). The mononuclear cells were isolated by percoll density gradient centrifugation (Sigma-Aldrich, St. Louis, MO, USA) at 2,000 *g* for 20 minutes. After three rinses, the isolated cells were cultured in endothelial basal medium-2 (EBM-2) with an MV Bullet Kit (Lonza, Walkersville, MD, USA). After 7 days of culture, the EPC markers DiI-labeled acetylated low-density lipoprotein (DiI-AcLDL) (Sigma-Aldrich) and bandeiraea simplicifolia lectin 1 (BS-1 lectin) (Sigma-Aldrich) were confirmed by immunofluorescence analysis.

### Preparation of lentivirus vectors and endothelial progenitor cell infection

The recombinant lentiviruses (Lenti) carrying murine CCR5 (Lenti-EGFP-CCR5) or a control transgenic EGFP (Lenti-EGFP) were prepared as previously described [19]. Passage 2 of EPCs was used for lentivirus transfection. Prior to transfection, cultured EPCs were labeled with CM-DiI (4 mg/mL; Molecular Probes, Eugene, OR, USA) for 15 minutes in accordance with the protocol of the manufacturer. Then the EPCs were incubated in EBM-2 containing Lenti-EGFP-CCR5 or Lenti-EGFP particles. The lentiviral particles were removed after 24 hours. The double-labeled EPCs were harvested (passage 3) and resuspended at 1 × 10^6^ cells/mL in PBS for administration to the mice.

### Experimental design

Sixty splenectomized ApoE^−/−^ mice were treated with a high-fat, high-cholesterol diet (21% anhydrous milk fat/butter fat, 34% sucrose and 0.2% cholesterol; Teklad; Harlan Laboratories, Indianapolis, IN, USA) for 24 weeks. Then the animals were randomly divided into three groups and intravenously injected with 200 μL of sterile PBS (control group) or 200 μL of 1 × 10^6^/mL EPCs transfected with Lenti-EGFP (Lenti-EGFP group) or 200 μL of 1 × 10^6^/mL EPCs infected with Lenti-EGFP-CCR5 (Lenti-CCR5 group). After treatment, all mice were returned to normal chow diet for the remaining experiments.

### Quantification and morphometry of atherosclerotic lipid deposition

After treatments, 15 mice in each group were sacrificed and aortas were isolated after saline perfusion to prepare frozen sections as described previously [13,15,17,18]. The plaque areas were measured in four subsequent sections for each mouse, and each section included three segments [18]. The images of the sections were captured with a digital camera (Canon, Tokyo, Japan) and analyzed by Image-Pro Plus Software (Media Cybernetics, Silver Spring, MD, USA). The aortic sinus frozen sections were stained with Oil Red O (Sigma-Aldrich), and the positive staining area was used to quantitate the atherosclerotic plaque area [19,20].

Human ascending aorta samples were obtained from the Department of Pathology of Qianfoshan Hospital. The plaques were classified for progression stages (early, advanced stable, and advanced unstable) as described previously [21]. All of the studies were performed in accordance with the ‘Code for Proper Secondary Use of Human Tissue’ and approved by the Ethical Committee of Shandong University. All donors provided written informed consent.

### Immunohistochemistry and immunofluorescence

Immunohistochemical assays were performed with the following primary antibodies: rabbit anti-α smooth muscle actin (1:150 dilution; Abcam, Cambridge, MA, USA), rat anti-Monocyte + Macrophage (MOMA-2, 1:100 dilution; Abcam), rat anti-CD31 (1:150 dilution; BD Biosciences, San Diego, CA, USA), goat anti-CCL5/RANTES (1:25 dilution; Santa Cruz Biotechnologies, Dallas, TX, USA), goat anti-CCR5 (1:25 dilution; Santa Cruz Biotechnologies), rabbit anti-IL-6 (1:500 dilution; Abcam), and rabbit anti-MMP9 (1:250 dilution; Abcam). Secondary antibodies used in these assays were purchased from Jackson ImmunoResearch Laboratories, Inc. (West Grove, PA, USA). The reaction was visualized by staining with 3, 3-diaminobenzidine (DAB) (Abcam). The ECs were identified by anti-CD31 antibody, and the staining intensity was evaluated as described previously [22]. The staining intensity is rated on a scale of 0 to 3: 0 = negative, 1 = weak, 2 = moderate, and 3 = strong. The accumulation of EPCs over the atherosclerotic plaques was evaluated by immunofluorescence analysis. In brief, double-labeled EPCs (EGFP and CM-DiI) were transplanted into the ApoE^−/−^ mice, and the mice were sacrificed on weeks 1 and 6 after transplantation. The aortic roots were excised, and 6-μm frozen sections were prepared as described previously [13,17,18]. The frozen sections were stained with 4′, 6-diamidino-2-phenylindole (DAPI) for 5 minutes. The number of triple-color positive cells (EGFP^+^, DiI^+^, and DAPI^+^) on atherosclerotic plaques was counted in three randomly chosen high-power fields (magnification, 200×), and representative results from three independent experiments are shown. To examine the incorporation of labeled putative EPCs into endothelium, the cross-sections of aortic roots of ApoE^−/−^ mice treated with EPCs were stained with primary rat anti-mouse CD31 (BD Biosciences, San Jose, CA, USA) or control rat IgG2a Ab (Abcam) and secondary donkey anti-rat Alexa Fluor 594 Ab (Life Technologies, Grand Island, NY, USA).

### Immunochemistry

Venous blood samples of the mice were collected 0, 1, and 6 weeks after treatment and centrifuged to collect the serum. Inflammatory cytokines were assessed by a mouse atherosclerosis antibody array I (RayBiotech Inc., Norcross, GA, USA), which consists of 22 antibodies for atherosclerosis-related cytokines. The Cluster version 3.0 and Java TreeView version 1.60 software (Michael Eisen’s lab) were used to analyze the results as described previously [23]. Fold change (FC) was used to identify genes with large shifts between the control group and treatment group [24]. Serum nitrite and nitrate were measured by a colorimetric nitric oxide (NO) metabolite detection kit (Cayman Chemical Company, Ann Arbor, MI, USA).

### Migration and proliferation assays

The migration of EPCs was evaluated by using transwell chambers (Costar Corp., Cambridge, MA, USA). In brief, mouse EPCs were resuspended at 5 × 10^4^ cells/100 μL in serum-free EBM-2 and seeded in the upper chambers. Recombinant mouse CCL5 (100 μg/mL; R&D Systems, Minneapolis, MN, USA), CCL5 (100 μg/mL) combined with anti-CCL5 antibody (6 μg/mL; Abcam), or EBM-2 was added into the lower chambers. After 24 hours, the number of EPCs migrated into lower chambers was counted manually in three randomly chosen high-power fields (magnification, 100×) per filter. The proliferation of EPCs was examined by using a 5-ethynyl-2′-deoxeuridine (EdU) assay kit (RiboBio Co., Ltd, Wuhan, China). EPCs were resuspended at 1 × 10^4^ cells/100 μL in serum-free EBM-2 and seeded in the 96-well plate (Costar Corp.) containing 50 nM EdU per well. After 72 hours, EPCs were fixed with 4% paraformaldehyde and subsequently incubated with 100 μL of 1× EdU staining liquid for 30 minutes. The DNA contents of EPCs were stained with DAPI (Sigma-Aldrich) for 5 minutes, and the EdU-positive cells were counted under a fluorescence microscope (IX-71; Olympus, Tokyo, Japan).

### Statistical analysis

Statistical differences between groups were examined by one-way analysis of variance with Mann-Whitney *U* test. A *P* value of less than 0.05 was considered statistically significant. All statistical analyses were performed by using SPSS 18.0 software (SPSS Inc., Chicago, IL, USA).

## Results

### Expression of CCL5 and CCR5 during progression of atherosclerotic plaques in humans and mice

**Figure 1:**
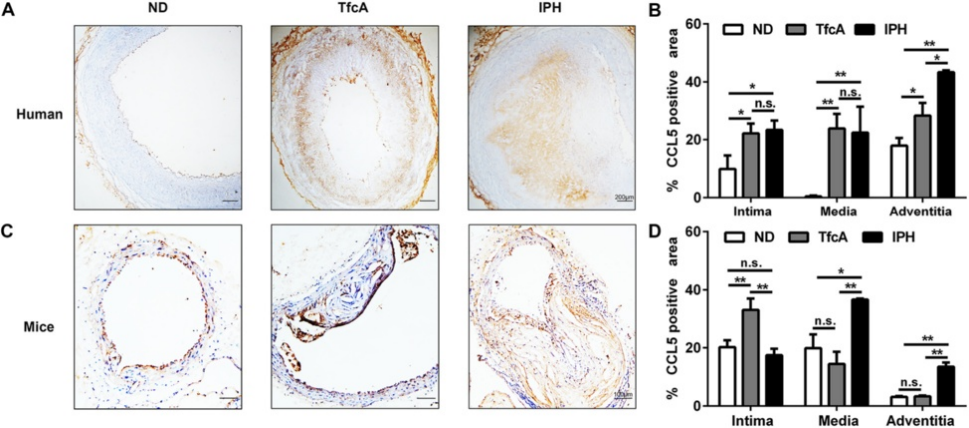
**CCL5 expression in ascending aorta of humans and mice. (A)** Immunohistochemistry of CCL5 in non-diseased artery, advanced plaques, and unstable plaques of human samples. **(B)** Quantitative analysis of CCL5 expression in (A) (n = 3, *P <0.05, **P <0.01). **(C)** Immunohistochemistry of CCL5 in non-diseased artery, advanced plaques, and unstable plaques of ApoE^−/−^ mice. **(D)** Quantitative analysis of CCL5 expression in (C) (n = 3, *P <0.05, **P <0.01). Bars represent mean ± standard deviation. ApoE, apolipoprotein E; CCL5, chemokine (C-C motif) ligand 5; IPH, thin capped fibroatheroma with intraplaque hemorrhage (advanced unstable plaques); ND, non-diseased arteries; n.s., non-significant; TfcA, thick fibrous cap atheroma (advanced stable plaques).

The expression of CCL5 in ascending atherosclerotic aorta of patients with atherosclerosis was examined by immunohistochemistry. As shown in Figure 1, the positive staining of CCL5 was observed in the arteries of the patients with no visible atherosclerotic plaques (intima, 9.93% ± 4.57%; media, 0.43% ± 0.29%; adventitia, 17.91% ± 2.66%), arteries of the patients with atherosclerotic plaques (intima, 22.18% ± 3.37%; media, 23.87% ± 5.04%; adventitia, 28.31% ± 4.42%), and arteries of the patients with advanced unstable plaques (intima, 23.38% ± 3.23%; media, 22.42% ± 9.02%; adventitia, 43.37% ± 0.67%). Quantitative image analysis indicated a significant increase of CCL5 expression in the intima and media of the patients with advanced unstable plaques in comparison with those with no visible atherosclerotic plaques (intima, *P* <0.05; media, *P* <0.01). However, no significant difference was found between the arteries of the patients with advanced unstable plaques and the arteries of the patients with atherosclerotic plaques (intima, *P* = 0.64; media, *P* = 0.81). CCL5 expression in the adventitia was significantly increased in the arteries of the patients with advanced unstable plaques in comparison with those with atherosclerotic plaques or no visible atherosclerotic plaques (*P* <0.05 and *P* <0.01, respectively).

CCL5 expression was also examined in ApoE^−/−^ mice fed a high-fat diet. As shown in Figure 1C, the positive staining area of CCL5 was observed in the arteries of the mice with no visible atherosclerotic plaques (intima, 20.23% ± 2.35%; media, 19.86% ± 4.75%; adventitia, 3.15% ± 0.30%), arteries of the mice with atherosclerotic plaques (intima, 33.09% ± 3.91%; media, 14.41% ± 4.21%; adventitia, 3.33% ± 0.30%), and arteries of the mice with advanced unstable plaques (intima, 17.43% ± 2.22%; media, 36.73% ± 0.45%; adventitia, 13.5% ± 1.41%). We found a significant decrease of CCL5 expression in the intima of the mice with advanced unstable plaques in comparison with those with atherosclerotic plaques (*P* <0.01). However, there were no significant differences between the arteries of the mice with advanced unstable plaques and the arteries of the mice with no visible atherosclerotic plaques (*P* = 0.20). CCL5 expression was significantly increased in the media of the mice with advanced unstable plaques in comparison with those with atherosclerotic plaques or no visible atherosclerotic plaques (*P <*0.01 and *P <*0.05, respectively). In the adventitia, the expression of CCL5 was significantly increased in the arteries of the mice with advanced unstable plaques in comparison with those with atherosclerotic plaques or no visible atherosclerotic plaques (*P <*0.01 and *P <*0.01, respectively). In addition, we examined the expression of CCR5 in ascending aorta of humans and mice. CCR5 protein was faintly detected in non-diseased artery and was highly expressed in the atherosclerotic plaques in humans and mice. (Additional file 1 shows this in more detail.)

### Overexpression of CCR5 increased endothelial progenitor cell recruitment to atherosclerotic plaques

**Figure 2:**
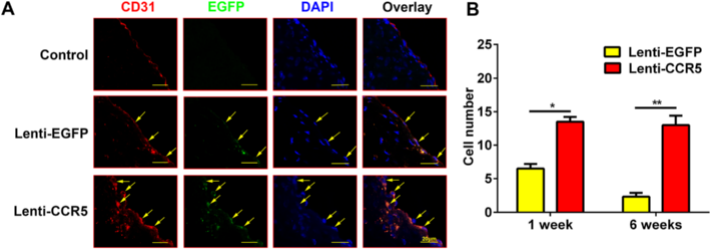
**Overexpression of CCR5 in endothelial progenitor cells (EPCs) promoted the recruitment of EPCs into atherosclerotic plaques. (A)** Representative images of EPC incorporation on atherosclerotic plaques 6 weeks after phosphate-buffered saline (PBS), EPC (Lenti-EGFP), and CCR5 overexpression of EPC treatment (Lenti-CCR5). DAPI (blue), EGFP (green), CD31 (red). Arrowheads indicate EPCs (EGFP^+^). **(B)** Quantitative analysis of migrated EPCs on atherosclerotic plaques in Lenti-EGFP and Lenti-CCR5 groups (*P <0.05, **P <0.01). The average number of EGFP^+^ EPCs on atherosclerotic plaques was counted from three randomly chosen high-power fields (magnification, 200×). Bars represent mean ± standard deviation. CCR5, chemokine (C-C motif) receptor 5; DAPI, 4′, 6-diamidino-2-phenylindole; EGFP, enhanced green fluorescent protein.

To investigate the effects of CCR5 overexpression on EPCs, CM-DiI-labeled EPCs were transfected by lentivirus encoded with EGFP (Lenti-EGFP) or EGFP-CCR5 (Lenti-CCR5) gene. The efficiency of the transfection in EPCs was evaluated by immunofluorescence, and more than 80% EPCs transfected with lentivirus vectors are positive for EGFP, indicating that EPCs with EGFP or EGFP-CCR5 expression were successfully established. (Additional file 2A shows this in more detail.) Western blot analysis displayed that the protein level of CCR5 was remarkably higher in the cells of the Lenti-CCR5 group than those of Lenti-EGFP group (Additional file 2). These transfected cells were intravenously injected into the ApoE^−/−^ mice treated with a high-fat diet for 24 weeks. To examine incorporation of labeled putative EPCs into endothelium, the cross-sections of aortic roots of ApoE^−/−^ mice treated with EPCs were stained with the antibody for CD31, the EC-specific marker. As shown in Figure 2, EPCs (EGFP^+^) were localized in the endothelium of the atherosclerotic plagues, suggesting that EPCs successfully engrafted in the vascular wall. EPCs (EGFP^+^) were also found in the sub-endothelial region of the atherosclerotic plagues in the Lenti-CCR5 group. In addition, the number of triple-color labeled EPCs detected in the atherosclerotic plaques of the Lenti-CCR5 group is significantly higher than that of the Lenti-EGFP group 1 week (13.5 ± 1.1 versus 6.5 ± 0.7, *P* <0.05) and 6 weeks post-treatment (13.0 ± 1.4 versus 2.3 ± 0.5, *P* <0.01), suggesting that CCR5 overexpression enhanced recruitment of EPCs over the atherosclerotic plaques.

Since EPCs may translocate into non-target organs of the mice after intravenous injection, we examined lung and liver to track the transplanted EPCs. Immunofluorescence staining showed that transplanted EPCs were detected in liver but not in lungs, and the numbers of EPCs recruited in the liver were similar between the Lenti-EGFP group and the Lenti-CCR5 group (data not shown).

### CCR5-overexpressing endothelial progenitor cell treatment enhanced stability of plaques

**Figure 3:**
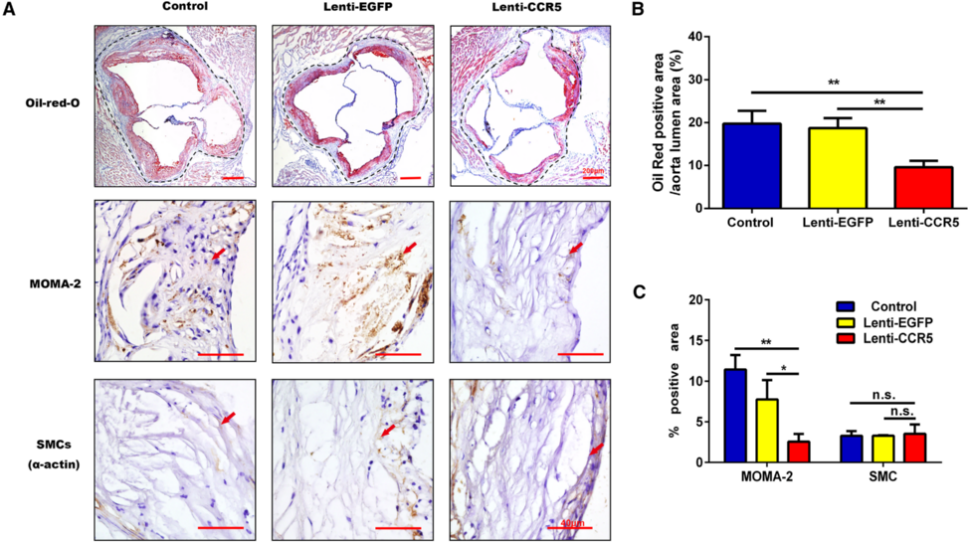
**The effects of CCR5-overexpressing endothelial progenitor cell treatment on plaque composition. (A)** Staining for the lipids, monocytes/macrophages (MOMA-2), and smooth muscle cells (SMCs) in control group, Lenti-EGFP group, and Lenti-CCR5 group of mice. Arrowheads indicate positive staining areas. **(B)** Ratio of Oil Red-positive staining area to aorta lumen area in control group, Lenti-EGFP group, and Lenti-CCR5 group of mice (n = 6, **P <0.01). **(C)** Quantitative analysis of the plaque composition of MOMA-2 and SMCs (n = 6, *P <0.05, **P <0.01). Bars represent mean ± standard deviation. CCR5, chemokine (C-C motif) receptor 5; EGFP, enhanced green fluorescent protein; n.s., non-significant.

To assess the effects of CCR5 overexpression EPC treatment on the stabilization of atherosclerotic plaques, we examined surrogate markers for plaque stability, including lipid burden, smooth muscle cells (SMCs), and monocyte/macrophage (MOMA-2) content in the plaques by immunostaining. Six weeks after the treatment, the plaques in the Lenti-CCR5 group (9.58% ± 1.51%) displayed a substantial regression in overall plaques area compared with the Lenti-EGFP (18.72% ± 2.52%) or the control group (19.75% ± 2.98%) as quantified by Oil Red O staining of the aortic sinus (Figure 3A, B). Quantitative image analysis showed a significant decrease in the positive staining area of MOMA-2 in the Lenti-CCR5 group (2.56% ± 0.94%) compared with the Lenti-EGFP group (7.76% ± 2.37%, *P <*0.05) or the control group (11.4% ± 1.78%, *P <*0.01). However, no significant difference was found for the plaque contents of SMCs between the Lenti-CCR5 (*P =* 0.76) or Lenti-EGFP (*P =* 0.97) group and the control group (Figure 3A, C). In summary, treatment with CCR5-overexpressing EPCs reduces lipid deposition and macrophage invasion in ApoE^−/−^ mice.

### CCR5-overexpressing endothelial progenitor cell treatment improved endothelial dysfunction of the atherosclerotic plaques

**Figure 4:**
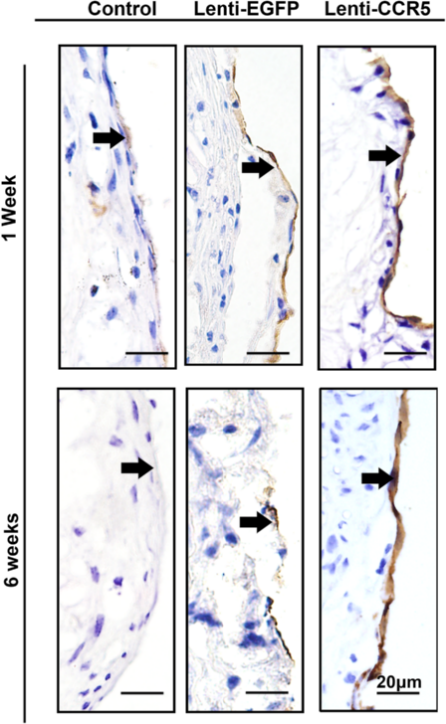
**CCR5-overexpressing endothelial progenitor cell treatment improved endothelial cell content. (A)** CD31 staining in coronary artery of control group, Lenti-EGFP group, and Lenti-CCR5 group of mice 1 week after treatment. **(B)** CD31 staining in coronary artery of control group, Lenti-EGFP group, and Lenti-CCR5 group of mice 6 weeks after treatment. Arrows refer to the endothelium of the luminal side of the coronary arteries. CCR5, chemokine (C-C motif) receptor 5; EGFP, enhanced green fluorescent protein.

**Figure 5:**
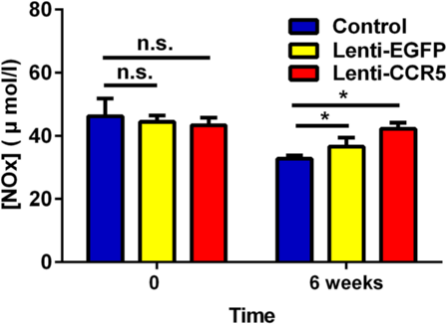
**CCR5-overexpressing endothelial progenitor cell treatment increased serum nitric oxide (NO).** The serum NO level of ApoE^−/−^ mice at baseline and 6 weeks after treatment with phosphate-buffered saline, Lenti-EGFP, and Lenti-CCR5 (n = 6, *P <0.05; n = 6). Bars represent mean ± standard deviation. ApoE, apolipoprotein E; CCR5, chemokine (C-C motif) receptor 5; EGFP, enhanced green fluorescent protein; n.s., non-significant.

Endothelial dysfunction is an independent predictor for atherothrombosis progression and is associated with plaque instability [25]. To investigate whether the CCR5-overexpressing EPC treatment improved the EC function in ApoE^−/−^ mice, we examined plaque content of ECs and the level of the serum NO, a molecule with protective effects in atherosclerosis. As shown in Figure 4, immunohistochemistry revealed a moderate to strong CD31^+^ staining in ascending arteries of the Lenti-CCR5 group on weeks 1 and 6 post-treatment. The CD31^+^ staining in ascending arteries of the Lenti-EGFP group was uniformly lower than in that of the Lenti-CCR5 group. In addition, serum NO level was remarkably higher in the Lenti-CCR5 group than in the Lenti-EGFP group as detected by colorimetric analysis (42.3 ± 1.8 versus 36.6 ± 2.8, *P* <0.05, Figure 5).

### CCR5-overexpressing endothelial progenitor cell treatment reduced pro-inflammatory factors in circulation and atherosclerotic plaques

**Figure 6:**
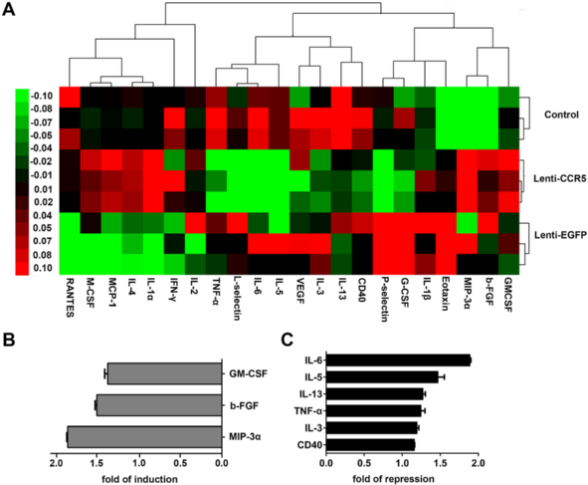
**Hierarchical clustering analysis of pro-inflammatory factors. (A)** The rows represent treatment groups, and the columns represent inflammatory factors. Red, black, and green colors indicate upregulation, unchanged expression, and downregulation compared with spike-in control. The color bars correspond to the relative expression level of inflammatory factors. **(B, C)** Fold changes were further analyzed for upregulation and downregulation of protein expression in (A). bFGF, basic fibroblast growth factor; EGFP, enhanced green fluorescent protein; G-CSF, granulocyte colony-stimulating factor; GM-CSF, granulocyte-macrophage colony-stimulating factor; IFN-γ, interferon-gamma; IL, interleukin; MCP-1, monocyte chemoattractant protein 1; M-CSF, macrophage colony-stimulating factor; MIP-3α, macrophage inflammatory protein-3α; RANTES, regulated on activation, normal T cell expressed and secreted; TNF-α, tumor necrosis factor alpha; VEGF, vascular endothelial growth factor.

Vascular inflammation is one of major causes of plaque rupture. We evaluated the serum levels of atherosclerosis-related inflammatory factors in mice treated with CCR5-overexpressing EPCs using mouse atherosclerosis antibody array I. Six weeks after treatment, nine proteins were identified with significant fold changes according to the heatmap diagram (Figure 6A). Among them, interleukin 6 (IL-6), IL-5, IL-3, IL-13, CD40, and tumor necrosis factor-alpha (TNF-α) were decreased in the Lenti-CCR5 group. Granulocyte-macrophage colony-stimulating factor (GM-CSF), macrophage inflammatory protein-3α (MIP-3α), and basic fibroblast growth factor (bFGF) were slightly increased in the Lenti-CCR5 group. The IL-6 level was declined by roughly 50% in the Lenti-CCR5 group compared with that in control group (Figure 6B, C). The plaque content of IL-6 was further examined by immunostaining.

**Figure 7:**
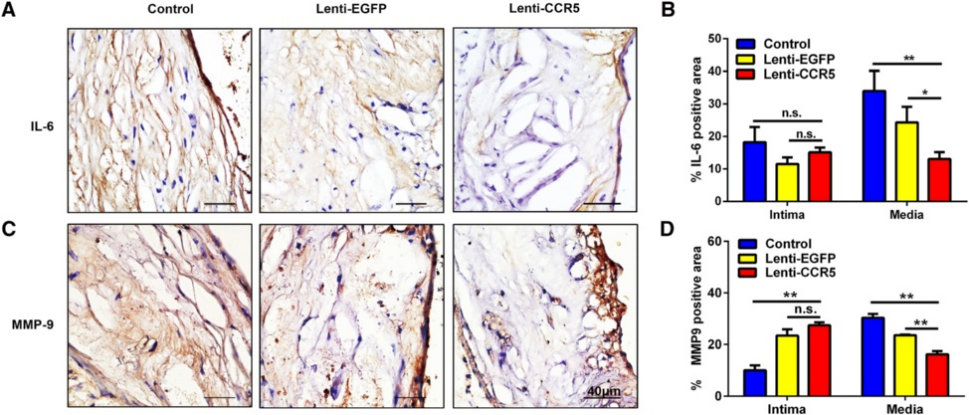
**CCR5-overexpressing endothelial progenitor cell treatment decreased interleukin-6 (IL-6) and matrix metalloproteinase 9 (MMP9) expression on the atherosclerotic plaques. (A)** Immunostaining of IL-6 on the aortic root plaques at 6 weeks after treatment with phosphate-buffered saline (PBS), Lenti-EGFP, or Lenti-CCR5. **(B)** Quantitative analysis of the IL-6 expression in (A) (n = 6, *P <0.05, **P <0.01). **(C)** Immunostaining of MMP9 on the aortic root plaques at 6 weeks after treatment with PBS, Lenti-EGFP, or Lenti-CCR5. **(D)** Quantitative analysis of the MMP9 expression in (C) (n = 6, *P <0.05, **P <0.01). Bars represent mean ± standard deviation. CCR5, chemokine (C-C motif) receptor 5; EGFP, enhanced green fluorescent protein; n.s., non-significant.

As shown in Figure 7A and B, the positive staining area of IL-6 in the media of arteries was significantly decreased in the Lenti-CCR5 group in comparison with that in the Lenti-EGFP or control group (Lenti-CCR5 versus Lenti-EGFP, 13.07% ± 2.11% versus 24.3% ± 4.85%, *P* <0.05; Lenti-CCR5 versus control, 13.07% ± 2.11% versus 34.03% ± 6.15%, *P* <0.01). However, no significant difference was found in the intima of arteries between the Lenti-CCR5 group (*P =* 0.34) or the Lenti-EGFP group (*P =* 0.06) and the control group. Matrix metalloproteinase 9 (MMP9) plays an essential role in local proteolysis of the extracellular matrix (ECM) [26]. Degradation of ECM components by MMP9 induces plaque inflammation and promotes plaque instability [27]. Thus, we examined the plaque content of MMP9 by immunostaining. As shown in Figure 7C and D, the positive staining area of MMP9 was significantly decreased in the media of arteries in the Lenti-CCR5 group in comparison with that in the Lenti-EGFP or control group (Lenti-CCR5 versus Lenti-EGFP, 16.26% ± 2.14% versus 23.57% ± 0.45%, *P* <0.01; Lenti-CCR5 versus control, 16.26% ± 2.14% versus 30.34% ± 2.57%, *P* <0.01). In the intima, there is no statistically significant difference between the Lenti-CCR5 and Lenti-EGFP groups (27.41% ± 1.92% versus 23.42% ± 4.58%, *P =* 0.23). However, significantly increased MMP9 expression was found in the Lenti-CCR5 group in comparison with that in the control group (27.41% ± 1.92% versus 10.07% ± 3.28%, *P* <0.01). Thus, MMP9 is increased in the intima but decreased in the media in atherosclerotic plaque with CCR5-overexpressing EPC treatment.

### CCL5/CCR5 interaction involved in endothelial progenitor cell migration

**Figure 8:**
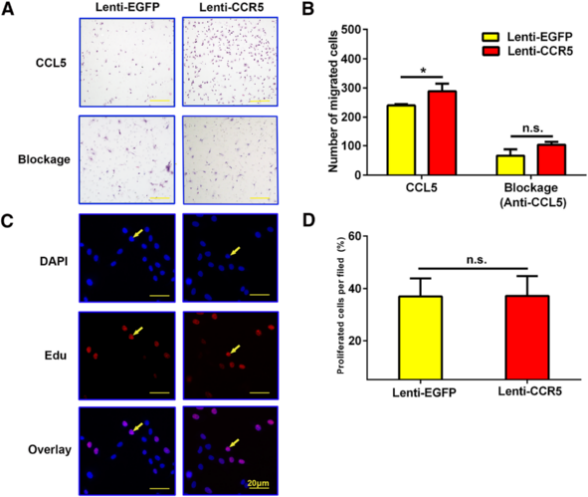
**Effects of CCL5 and CCR5 interaction on endothelial progenitor cell (EPC) migration and proliferation. (A)** Transwell migration assay of EPCs after transfection with Lenti-EGFP or Lenti-CCR5. **(B)** Quantitative analysis of the results in (A) (n = 3, *P <0.05, **P <0.01). **(C)** Representative images of 5-ethynyl-2′-deoxeuridine (EdU) cell proliferation after transfection with Lenti-EGFP or Lenti-CCR5. Arrows indicate EdU-positive cells. **(D)** Rate of EdU-positive cells in (C) (n = 3). Bars represent mean ± standard deviation. CCL5, chemokine (C-C motif) ligand 5; CCR5, chemokine (C-C motif) receptor 5; DAPI, 4′, 6-diamidino-2-phenylindole; EGFP, enhanced green fluorescent protein; n.s., non-significant.

Previous study showed that CCL5 significantly induced cell migration [16]. To confirm whether CCL5 interacts with CCR5 to promote the migration of EPCs, we performed *in vitro* chemotaxis assays with CCR5-overexpressing EPCs. As seen in Figure 8, CCL5 significantly enhanced the migration of EPCs in the Lenti-CCR5 group in comparison with the Lenti-EGFP group (*P* <0.01). After the addition of anti-CCL5 antibody, the numbers of migrated EPCs in the Lenti-EGFP group and Lenti-CCR5 group were similar (*P =* 0.04), suggesting that CCR5 overexpression induced EPC migration operates in a CCL5-dependent manner. Furthermore, we examined EPC proliferation by EdU incorporation (Figure 8C, D), and the cell growth was similar between the Lenti-CCR5 group and the Lenti-EGFP group (*P* = 0.98). Thus, overexpression of CCR5 promotes migration of EPCs without affecting their proliferation.

## Discussion

Chemokines are crucial for atherosclerotic progress [13,28,29]. In this study, we found that chemokine CCL5 was upregulated in advanced atherosclerotic plaques and generated EPCs overexpressing CCR5. These CCR5-overexpressing EPCs were injected into ApoE^−/−^ mice and induced a phenotype of more stable atherosclerotic plaques in these mice. In addition, we found that overexpression of CCR5 in EPCs contributes to endothelial repair and reduces pro-inflammatory cytokines.

Circulating EPCs may differentiate *ex vivo* into ECs [7,30] and improve EC regeneration in a variety of arterial injuries [31-34]. However, the mechanisms of EPC recruitment to the injury sites are not clear [35,36]. The chemokine receptor CCR5 is detected in EPCs, and CCR5-mediated signals may be involved in EPC recruitment from circulation to injured vascular wall [14,16]. In this study, we created a Lentivirus expression system to overexpress CCR5 in EPCs and injected these cells into ApoE^−/−^ mice. We demonstrated that CCR5 overexpression increased the number of EPCs recruited to atherosclerotic plaques and that CCR5-overexpressing EPCs enhanced the plaque stability. These observations indicated that CCR5 is involved in EPC recruitment and the stabilization of atherosclerotic plaques. In addition to CCL5/CCR5, other chemokines stabilize atherosclerotic plaques. For example, CXCL12 promotes the stabilization of atherosclerotic plaques by increasing the recruitment of smooth muscle progenitor cells to the plaques in ApoE^−/−^ mice [37]. The CXCL1-CXCR2 axis is required for the recruitment of BM EPCs to the plaques [19,38]. Taken together, specific chemokines mediate mobilization of progenitor cells to sites of atherosclerotic plaques and promote plaque stability.

EC function is important for atherothrombosis progression [3]. Endothelium regulates vessel wall relaxation, thrombosis, and homeostasis by production of NO [25,39]. Disturbance in NO production facilitates the aggregation of circulating monocytes/macrophages into sub-endothelial space and aggravates the instability of atherosclerotic plaques [40]. In this study, we found that CCR5-overexpressing EPCs increased the plaque content of ECs and the serum level of NO. This may result from enhanced EPC recruitment to the atherosclerotic plaques. The incorporation of EPCs may develop into ECs, increase the production of NO, and promote EC regeneration [19,40,41].

Vascular inflammation is involved in the processes of atherosclerosis and directly induces EC dysfunction [3,19]. In this study, we found that CCR5-overexpressing EPCs significantly reduced expression of IL-6 and MMP9 in the atherosclerotic plaques. CCR5 overexpression may suppress the production of IL-6 by restoring of the balance of T helper 17 cells (Th_17_) and regulatory T cells (Tregs) [18,42]. Th_17_/Tregs balance plays a fundamental role in the maintenance of the stability of atherosclerotic plaques [42,43]. The imbalance of Th_17_/Tregs aggravates the burden of plaques by modulation of IL-6 production and increases the risk of plaque rupture [42,43]. Recent study reported that CCR5 tightly regulates Th_17_/Tregs balance by modulating the sphingosine 1-phosphate receptor (S1PR)-dependent T-cell egress process [44-47]. Therefore, administration with CCR5-overexpressing EPCs may contribute to the restoration of the Th_17_/Tregs balance and diminish inflammatory responses. In the present study, CCR5-overexpressing EPCs enhanced the EPC recruitment to the atherosclerotic plaques. Since the mobilizers of progenitor cells such as granulocyte colony-stimulating factor (G-CSF) significantly decreased the levels of MMP9 in atherosclerotic mice [18], increased EPC recruitment may also suppress MMP9 expression in the atherosclerotic plaques.

It has been shown that specific interactions between chemokines and their receptors mediate the chemotaxis activation of EPCs [12,14,48]. Our *in vitro* study demonstrated that CCL5-induced EPC migration was increased by overexpression of CCR5 and that the increase was abolished by the addition of CCL5 antibody, suggesting that CCL5/CCR5 interaction is involved in chemotactic effects of EPCs*.* This is consistent with previous studies that CCL5 directly induced EPC migration as both [^44^AANA^47^]-RANTES/CCL5 and [E66A]-RANTES/CCL5 mutants displayed a reduced ability to induce the migration of EPCs [15]. CCL5 is highly expressed in atherosclerotic plaques. Overexpression of CCR5 might promote migration of EPC toward tissues with higher expression of CCL5 and thus enhance EPC recruitment to the atherosclerotic plaques.

## Conclusions

In summary, we provided evidence for improving atherosclerotic plaque stability through treatment of CCR5-overexpressing EPCs. If CCR5 has a similar function in humans, stimulation of CCR5 expression in EPCs may promote EPC recruitment, restore EC function, decrease pro-inflammatory factors, and eventually improve the stabilization of atherosclerotic plaques.

## Additional files

Additional file 1:
**Analysis of CCR5 expression in ascending aorta of humans and mice. (A)** Representative images of immunostaining of CCR5 in non-diseased arteries, advanced plaques, and unstable plaques in human. **(B)** Quantitative analysis of CCR5 expression in (A) (n = 3, **P* <0.05, ***P* <0.01). **(C)** Representative images of immunostaining of CCR5 in non-diseased arteries, advanced plaques, and unstable plaques in ApoE^−/−^ mice. **(D)** Quantitative analysis of CCR5 expression in (C) (n = 3, **P* <0.05, ***P* <0.01). Bars represent mean ± standard deviation (SD). ApoE, apolipoprotein E; CCL5, chemokine (C-C motif) ligand 5; IPH, thin capped fibroatheroma with intraplaque hemorrhage (advanced unstable plaques); ND, non-diseased arteries; n.s., non-significant; TfcA, thick fibrous cap atheroma (advanced stable plaques). Additional file 2:
**The efficiency of lentivirus infection in endothelial progenitor cells (EPCs). (A)** Enhanced green fluorescent protein (EGFP) fluorescence examination of EPCs 72 hours after transfection with Lenti-EGFP and Lenti-EGFP-CCR5 vector. Arrowheads indicate EGFP-positive cells. Scale bar = 40 μm. **(B)** Immunoblots of CCR5 and β-actin of extracts from EPCs transfected with Lenti-EGFP or Lenti-CCR5.

## Abbreviations

ApoE: apolipoprotein E
BM: bone marrow
CCL5: chemokine (C-C motif) ligand 5
CCR5: chemokine (C-C motif) receptor 5
DAPI: 4′, 6-diamidino-2-phenylindole
EBM-2: endothelial basal medium-2
EC: endothelial cell
ECM: extracellular matrix
EdU: 5-ethynyl-2′-deoxeuridine
EGFP: enhanced green fluorescent protein
EPC: endothelial progenitor cell
IL: interleukin
MMP9: matrix metalloproteinase 9
MOMA-2: monocyte/macrophage
NO: nitric oxide
PBS: phosphate-buffered saline
RANTES: regulated on activation, normal T cell expressed and secreted
SMC: smooth muscle cell
Th_17_: T helper 17 cell
Treg: regulatory T cell
