# Supplementary figures and images for: CCR5 facilitates endothelial progenitor cell recruitment and promotes the stabilization of atherosclerotic plaques in ApoE−/− mice
Source: Stem Cell Res Ther. 2015 Mar 19;6(1):36. doi: 10.1186/s13287-015-0026-0 (PMC4404610; doi:10.1186/s13287-015-0026-0)

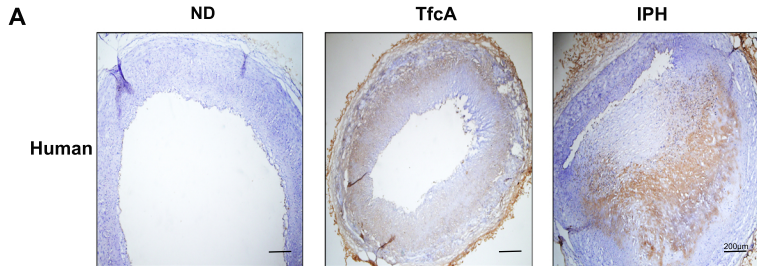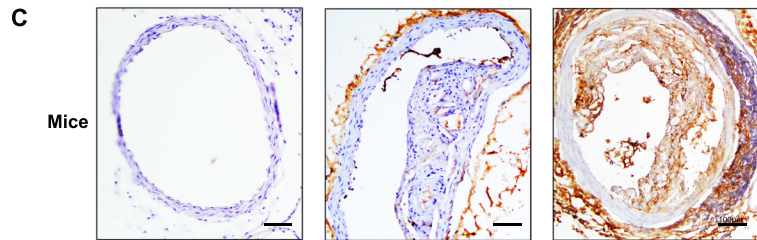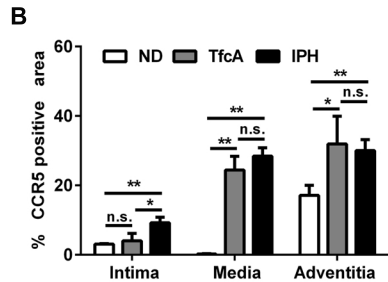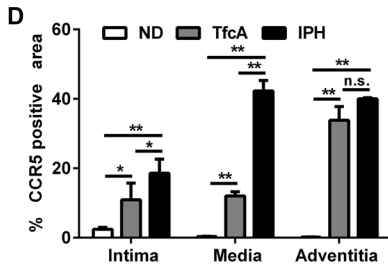

Supplement: Additional file 1: — Analysis of CCR5 expression in ascending aorta of humans and mice. (A) Representative images of immunostaining of CCR5 in non-diseased arteries, advanced plaques, and unstable plaques in human. (B) Quantitative analysis of CCR5 expression in (A) (n = 3, *P <0.05, **P <0.01). (C) Representative images of immunostaining of CCR5 in non-diseased arteries, advanced plaques, and unstable plaques in ApoE−/− mice. (D) Quantitative analysis of CCR5 expression in (C) (n = 3, *P <0.05, **P <0.01). Bars represent mean ± standard deviation (SD). ApoE, apolipoprotein E; CCL5, chemokine (C-C motif) ligand 5; IPH, thin capped fibroatheroma with intraplaque hemorrhage (advanced unstable plaques); ND, non-diseased arteries; n.s., non-significant; TfcA, thick fibrous cap atheroma (advanced stable plaques). [file 13287_2015_26_MOESM1_ESM.pdf]

**A****Lenti-EGFP**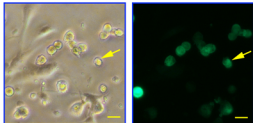**Lenti-CCR5**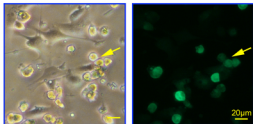**B**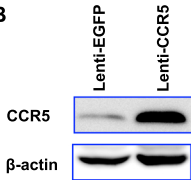

Supplement: Additional file 2: — The efficiency of lentivirus infection in endothelial progenitor cells (EPCs). (A) Enhanced green fluorescent protein (EGFP) fluorescence examination of EPCs 72 hours after transfection with Lenti-EGFP and Lenti-EGFP-CCR5 vector. Arrowheads indicate EGFP-positive cells. Scale bar = 40 μm. (B) Immunoblots of CCR5 and β-actin of extracts from EPCs transfected with Lenti-EGFP or Lenti-CCR5. [file 13287_2015_26_MOESM2_ESM.pdf]
